# Colokinetic effect of noradrenaline in the spinal defecation center: implication for motility disorders

**DOI:** 10.1038/srep12623

**Published:** 2015-07-28

**Authors:** Kiyotada Naitou, Takahiko Shiina, Kurumi Kato, Hiroyuki Nakamori, Yuuki Sano, Yasutake Shimizu

**Affiliations:** 1 Department of Basic Veterinary Science, Laboratory of Physiology, The United Graduate School of Veterinary Sciences, Gifu University, 1-1 Yanagido, Gifu 501-1193, Japan

## Abstract

Chronic abdominal pain in irritable bowel syndrome (IBS) usually appears in combination with disturbed bowel habits, but the etiological relationship between these symptoms remains unclear. Noradrenaline is a major neurotransmitter controlling pain sensation in the spinal cord. To test the hypothesis that the descending noradrenergic pathway from the brain stem moderates gut motility, we examined effects of intrathecal application of noradrenaline to the spinal defecation center on colorectal motility. Colorectal intraluminal pressure and expelled volume were recorded *in vivo* in anesthetized rats. Intrathecal application of noradrenaline into the L6-S1 spinal cord, where the lumbosacral defecation center is located, caused propulsive contractions of the colorectum. Inactivation of spinal neurons by tetrodotoxin blocked the effect of noradrenaline. Pharmacological experiments showed that the effect of noradrenaline is mediated primarily by alpha-1 adrenoceptors. The enhancement of colorectal motility by intrathecal noradrenaline was abolished by severing of the pelvic nerves. Our results demonstrate that noradrenaline acting on sacral parasympathetic preganglionic neurons through alpha-1 adrenoceptors causes propulsive motility of the colorectum in rats. Considering that visceral pain activates the descending inhibitory pathways including noradrenergic neurons, our results provide a rational explanation of the concurrent appearance of chronic abdominal pain and colonic motility disorders in IBS patients.

Chronic abdominal pain or discomfort is a major symptom of functional gastrointestinal disorders, such as irritable bowel syndrome (IBS). The symptom is thought to be related to visceral hypersensitivity. In line with this, it has been reported that IBS patients exhibit lowered colorectal pain thresholds to mechanical stimuli^1^. The visceral hypersensitivity is postulated to be brought about by sensitization of the primary sensory afferents and spinal ascending neurons, both of which are involved in the transmission of visceral sensation to the supraspinal level^2^. In addition to chronic abdominal pain or discomfort, altered motility of the gut is a defining symptom of IBS^3^. The motility alteration could contribute to chronic abdominal pain when the gut becomes hypersensitive. Conversely, hypersensitivity could alter motility through overactive neural reflexes. Thus, it is probable that these concurrently appearing symptoms impact each other^2^,^4^. However, a causal relationship between hypersensitivity and altered motility remains unclear.

Nociceptive stimuli, including visceral pain, activate a descending inhibitory pathway from the brain stem, by which noxious input is suppressed in the spinal cord^5^,^6^. One of the major components of the descending inhibitory pathway is noradrenergic neurons derived from the pons, especially A5, 6 and 7 regions^5^,^7^,^8^ In fact, experimental noxious stimuli have been shown to increase the release of noradrenaline (NA) in the spinal cord in rats^9^,^10^. The concurrent appearance of hypersensitivity and altered motility prompted us to hypothesize that enhancement of NA release in the spinal cord via activation of the descending inhibitory pathway causes altered motility. In support of this hypothesis, Takaki *et al.* demonstrated that electrical stimulation of the pons facilitates rectal contraction in guinea-pigs^11^, suggesting the presence of a descending pathway connecting between the pons and gut with a relay in the spinal cord.

The aim of this study was to test the hypothesis that descending noradrenergic pathways from the brain stem could moderate gut motility. Validation of this hypothesis would provide a new insight into the etiology of IBS. For this purpose, we used an *in vivo* experimental system for measurement of colorectal motility and examined the effects of intrathecally applied NA at the L6-S1 level of the spinal cord, where the lumbosacral defecation center is located in rats. Our results clearly showed that NA acting at the lumbosacral spinal cord causes strong propulsive motility of the colorectum through mediation of the pelvic nerves.

## Results

### Effects of noradrenaline injected into the lumbosacral spinal cord

**Figure 1:**
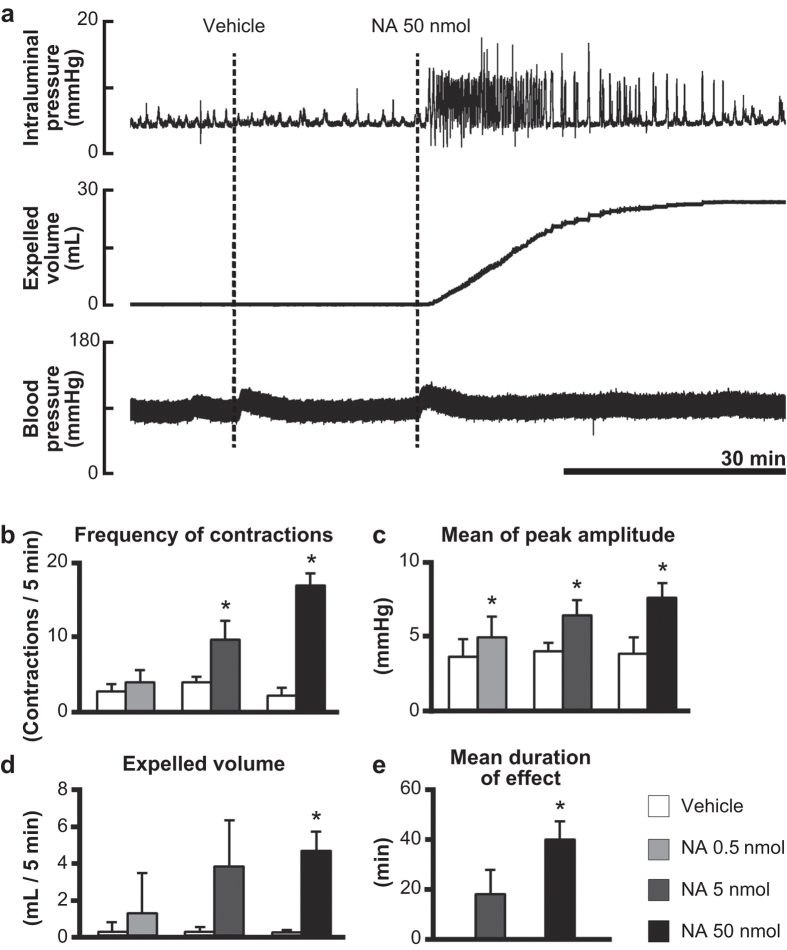
Responses of the colorectum to intrathecal noradrenaline applied to the lumbosacral spinal cord. (**a**) Representative recording traces of intraluminal pressure change (upper), expelled liquid volume (middle) and blood pressure (lower) after intrathecal application of NA (50 nmol) are shown. Vehicle denotes time of 0.9% saline (10 μL) injection as a control. Bar graphs summarize (**b**) frequency of contractions (contractions/5 min), (**c**) mean of peak amplitude (mmHg), and (**d**) expelled liquid volume (mL/5 min) after saline injection and after NA (0.5, 5 and 50 nmol) injection. (**e**) Mean duration of enhanced motility (min) after injection of NA (5 and 50 nmol) is shown. Each value represents the mean ± SD (n = 4). *Significantly different from values of vehicle control (*P* < 0.05).

At a resting state before NA application, brief and small rises in intraluminal pressure, representing changes in contractile activity of the colorectum, occurred spontaneously without accompanying fluid output from the anal cannula (Fig. 1a). When 0.9% saline was injected into the lumbosacral spinal cord as a control, little or no change in colorectal motility was observed (frequency, 2.3 ± 0.96 contractions/5 min; amplitude, 3.9 ± 1.1 mmHg; expelled volume, 0.1 ± 0.1 mL/5 min). Subsequent injection of NA at a dose of 0.5 nmol had no effect on colorectal motility (frequency, 4.0 ± 1.8 contractions/5 min, *P* > 0.05; amplitude, 4.9 ± 1.5 mmHg, *P* < 0.05; expelled volume, 1.3 ± 2.2 mL/5 min, *P* > 0.05), whereas injection at a dose of 5 or 50 nmol significantly increased the frequency of colorectal contractions (5 nmol, 9.8 ± 2.5 contractions/5 min, *P* < 0.05; 50 nmol, 17.0 ± 1.8 contractions/5 min, *P* < 0.05), the amplitude of colorectal contractions (5 nmol, 6.4 ± 1.1 mmHg, *P* < 0.05; 50 nmol, 7.6 ± 0.97 mmHg, *P* < 0.05) and the expelled fluid volume (5 nmol, 3.8 ± 2.5 mL/5 min, *P* > 0.05; 50 nmol, 4.6 ± 1.1 mL/5 min, *P* < 0.05) as shown in Fig. 1. The frequency and amplitude significantly increased in a dose dependent manner (*P* < 0.05). Also, the mean duration of enhanced motility was significantly prolonged as the injected dose of NA was increased (Fig. 1e, 5 nmol, 17.9 ± 7.3 min vs. 50 nmol, 39.4 ± 9.7 min, *P* < 0.05). These results indicate that intrathecal application of NA triggers propulsive contractions in the colorectum. Intravenous injection of atropine (1 mg/kg bolus i.v. and 6 mg/kg/h maintenance infusion i.v.) inhibited the effect of NA on colorectal motility (data not shown), suggesting that the final mediator to induce smooth muscle contraction is acetylcholine released from the enteric neurons. Although a transient increase in blood pressure was observed after injection of saline or NA, intrathecal NA did not exert any specific effect on blood pressure (Fig. 1a).

### Effects of tetrodotoxin on noradrenaline-enhanced colorectal motility

**Figure 2:**
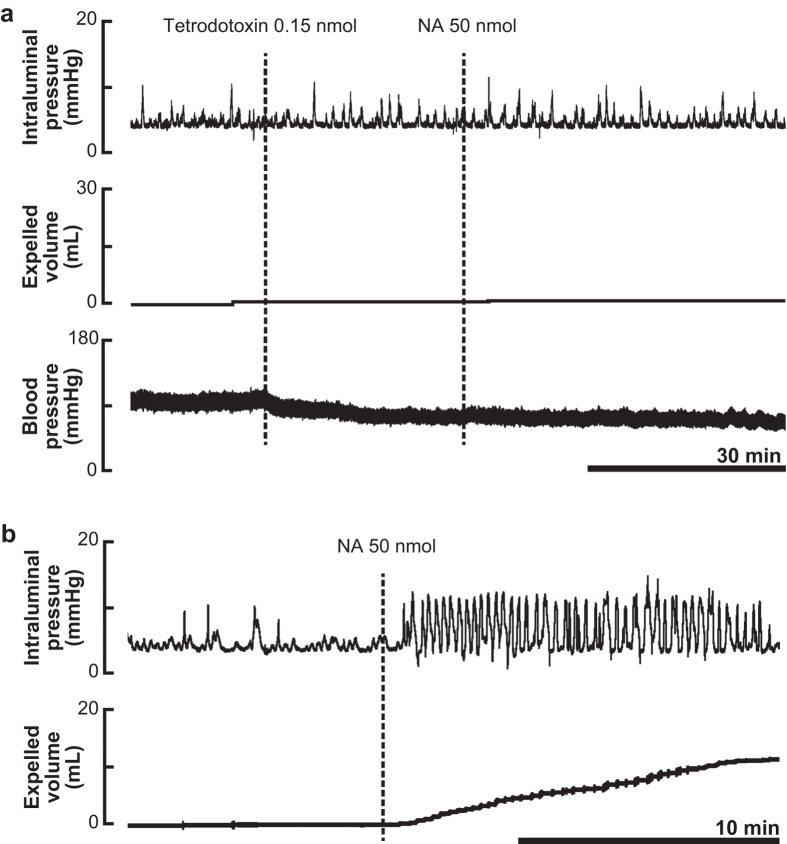
Effects of pretreatment with tetrodotoxin or transection of the thoracic spinal cord at T8 on the colokinetic effect of noradrenaline. (**a**) Representative recording traces of intraluminal pressure change (upper), expelled liquid volume (middle) and blood pressure (lower) before and after injection of NA (50 nmol) under the condition of tetrodotoxin-induced neural inactivation are shown. (**b**) Representative recording traces of intraluminal pressure change (upper) and expelled liquid volume (lower) before and after injection of NA (50 nmol) under the condition of neural disconnection between the brain stem and spinal defecation center by transection of the thoracic spinal cord (T8 level) are shown. Similar results were reproducibly obtained in three independent rats in both experiments.

To examine whether the action of NA is neurogenic, the neuronal blocker tetrodotoxin was intrathecally applied prior to injection of NA. Tetrodotoxin (0.15 nmol, i.t. at the L6-S1 spinal level) caused no apparent changes in spontaneous contractions of the colorectum but reduced blood pressure (Fig. 2a). Under the condition in which neurons of the lumbosacral spinal cord were inactivated by intrathecal administration of tetrodotoxin, NA failed to enhance colorectal motility (Fig. 2a).

### Effects of surgical transection of the thoracic spinal cord at T8 on the colokinetic effect of noradrenaline

To examine whether activity of supraspinal regions is essential for the action of NA, the thoracic spinal cord was transected at the T8 level. One hour after transection of the thoracic spinal cord, 50 nmol of NA was injected into the L6-S1 spinal cord. As shown in Fig. 2b, NA caused a marked propulsive motility of the colorectum, which is comparable to that observed in rats without transection of the thoracic spinal cord (cf. Fig. 1a).

### Pharmacological characterization of adrenoceptor subtypes that mediate the action of noradrenaline

**Figure 3:**
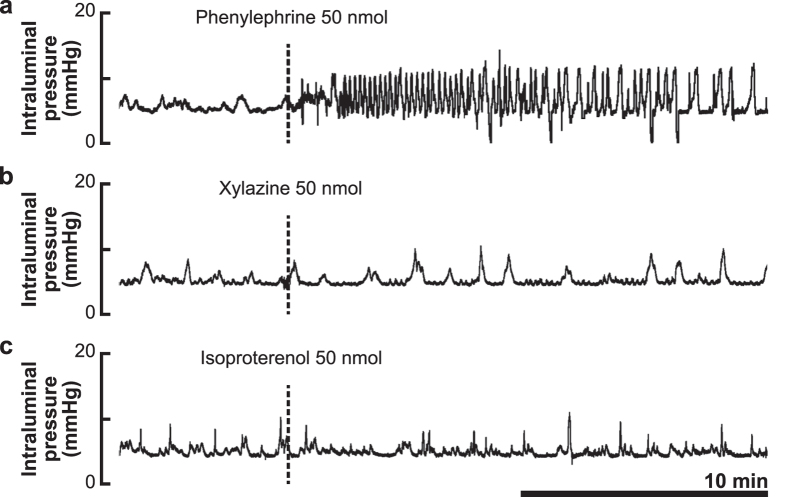
Effects of adrenoceptor subtype-selective agonists applied to the lumbosacral spinal cord. Representative recording traces of intraluminal pressure change before and after intrathecal application of the alpha-1 adrenoceptor agonist phenylephrine (50 nmol), alpha-2 adrenoceptor agonist xylazine (50 nmol) and beta adrenoceptor agonist isoproterenol (50 nmol) are shown in panels **a**–**c**, respectively. Similar results were reproducibly obtained in three independent rats in each experiment.

The alpha-1 adrenoceptor agonist phenylephrine (50 nmol, i.t.) mimicked the colokinetic effect of NA (Fig. 3a). In contrast, both the alpha-2 adrenoceptor agonist xylazine (50 nmol, i.t.) and the beta adrenoceptor agonist isoproterenol (50 nmol, i.t.) had no effect on colorectal motility, as shown in Fig. 3b,c, respectively.

**Figure 4:**
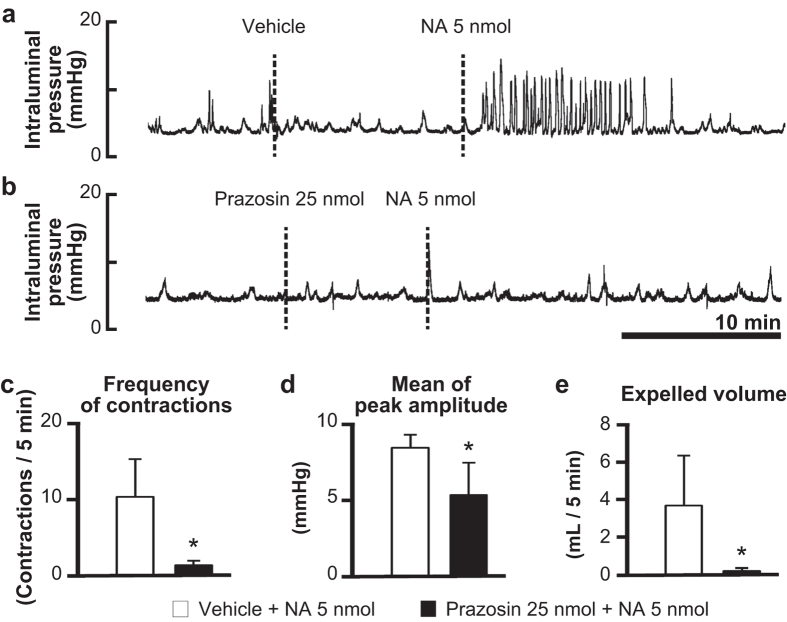
Effects of alpha-1 adrenoceptor antagonist on the colokinetic effect of noradrenaline. Representative recording traces of intraluminal pressure change in response to intrathecal injection of NA (5 nmol) with vehicle or alpha-1 adrenoceptor antagonist prazosin (25 nmol) pretreatment are shown in panels **a** and **b**, respectively. Bar graphs summarize (**b**) frequency of contractions (contractions/5 min), (**c**) mean of peak amplitude (mmHg) and (**d**) expelled liquid volume (mL/5 min) after 5 nmol of NA injection in the absence or presence of the antagonist. Each value represents the mean ± SD (n = 4). *Significantly different from values of vehicle control (*P* < 0.05).

Intrathecal injection of the alpha-1 adrenoceptor antagonist prazosin (25 nmol) alone had no effect on spontaneous colorectal contractions, but the antagonist completely inhibited the action of subsequently applied NA (5 nmol, i.t.) (Fig. 4). Since prazosin is a competitive antagonist, we used a lower dose of NA (5 nmol) in this experiment. Quantitative analyses showed that prazosin significantly diminished all parameters of NA-induced responses compared with those after vehicle injection (frequency, 1.5 ± 0.6 vs. 10.3 ± 5.1 contractions/5 min, *P* < 0.05; amplitude, 5.3 ± 2.1 vs. 8.4 ± 0.8 mmHg, *P* < 0.05; expelled volume, 0.03 ± 0.05 vs. 3.8 ± 2.7 mL/5 min, *P* < 0.05) (Fig. 4c–e).

### Effects of surgical nerve transections on the noradrenaline-induced enhancement of colorectal motility

**Figure 5:**
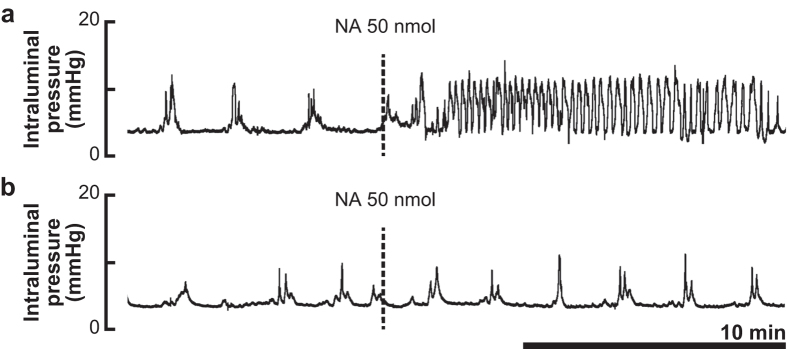
Effects of surgical nerve transections on the colokinetic effect of noradrenaline. Representative recording traces of intraluminal pressure change in response to intrathecal injection of NA (50 nmol) after severing the lumbar colonic nerve (**a**) or the pelvic nerve (**b**) are shown. Similar results were reproducibly obtained in three independent rats in each experiment.

The colorectum receives efferent innervation from the lumbosacral spinal cord including the sympathetic lumbar colonic nerves and parasympathetic pelvic nerves. We therefore transected these nerves to identify the nerve pathway that mediates the action of NA. Transection of the lumbar colonic nerves did not affect the NA-induced enhancement of colorectal motility (frequency, 16.0 ± 4.6 contractions/5 min; amplitude, 7.6 ± 0.6 mmHg; expelled volume, 4.0 ± 1.0 mL/5 min, Fig. 5a). On the other hand, when the pelvic nerves were bilaterally transected, intrathecal NA failed to increase the frequency of contractions or amplitude of colorectal pressure changes (Fig. 5b). Similarly, the expelled fluid volume was not increased by NA injection after the nerve transection (data not shown).

## Discussion

The present study was undertaken to test our hypothesis that the noradrenergic descending pathway from the brain stem moderates gut motility by acting on the lumbosacral spinal defecation center. Our principal findings were: 1) intrathecal application of NA to the L6-S1 region of the spinal cord caused strong propulsive motility of the colorectum, 2) the effect of NA was abolished by blocking neurons at the L6-S1 spinal cord with tetrodotoxin, and 3) NA-induced enhancement of colorectal motility was observed even after transection of the thoracic spinal cord but was abolished by cutting the pelvic nerves. In addition, pharmacological experiments with subtype-selective adrenoceptor agonists and antagonists revealed that the prokinetic effect of NA was mediated primarily by alpha-1 adrenoceptors. These results indicate that NA activates parasympathetic preganglionic neurons of the lumbosacral spinal cord through activation of alpha-1 adrenoceptors, resulting in enhanced colorectal motility. Considering that nociceptive stimuli including visceral pain activate the descending inhibitory pathway to suppress noxious input in the spinal cord^5^,^6^, our results provide a rationale for the concurrent appearance of chronic abdominal pain and colonic dysmotility in IBS patients.

Intrathecal injection of tetrodotoxin appears to inactivate spinal neurons locally^12^, as the drug decreased blood pressure (Fig. 2) but did not cause respiratory change (data not shown). The fact that NA failed to enhance colorectal motility after treatment with tetrodotoxin indicates that the action of NA in the lumbosacral spinal cord is neurogenic. It could be possible to deduce that neurons directly activated by NA are located in supraspinal regions because intrathecally injected NA would be delivered to the defecation center in the brain stem through cerebrospinal fluid circulation. However, our data show this is not the case. As shown in Fig. 2a, transection of the thoracic spinal cord at the T8 level did not abolish the effects of NA. Since the transection blocks connections between the supraspinal regions and the lumbosacral spinal cord, our results indicate that the site of action of NA is at the lumbosacral spinal cord.

Our data obtained by pharmacological experiments revealed that the receptor subtype mediating the prokinetic effect of NA is alpha-1 adrenoceptors. Regional distribution studies with *in situ* hybridization have shown that alpha-1 adrenoceptor mRNAs are abundantly expressed in the motor neurons and intermediolateral cell column of the lumbosacral spinal cord in rats^13^ and human^14^. The intermediolateral cell column is located at the middle of the grey matter of the spinal cord and consists of a mass of preganglionic neural cells of the sympathetic or parasympathetic nerves. It has been demonstrated that stimulation of alpha-1 adrenoceptors activates GABAergic and glycinergic inhibitory interneurons in the dorsal horn of the lumbosacral spinal cord of rats^15^,^16^. This allows us to speculate that intrathecal NA binds to alpha-1 adrenoceptors on the interneurons and indirectly suppresses the activity of sympathetic preganglionic neurons. The suppression of sympathetic nerve activity can lead to enhancement of colorectal motility by removing tonic inhibitory influences^17^,^18^. However, this seems unlikely given the fact that surgical cutting of the sympathetic colonic nerves failed to block the effects of NA (Fig. 5a). Alternatively, it is possible that NA activates sacral parasympathetic preganglionic neurons directly acting on their alpha-1 adrenoceptors. In support of this, we found that transection of the parasympathetic pelvic nerve prevents the colokinetic effect of NA (Fig. 5b).

IBS is a functional disorder of the gastrointestinal tract characterized by abdominal pain or discomfort in the absence of a structural basis for pathogenesis^19^. Although the symptoms usually appear in combination with disturbed bowel habits^3^,^4^, the etiological relationship between these symptoms remains unclear. Based on the current evidence, a number of hypotheses have been formulated about the mechanisms of visceral hypersensitivity^20^. These include (1) the sensitization of peripheral visceral afferent neurons; (2) the sensitization of spinal cord dorsal horn neurons; (3) the altered descending excitatory and inhibitory influences to the spinal cord nociceptive neurons; and (4) the misinterpretation of innocuous sensation as noxious due to cognitive and emotional biasing^20^. However, it is still unclear that the relationship between these sensitization and dysmotility of the gut. In the present study, we demonstrated that NA acting on the lumbosacral defecation center promotes substantial enhancement of colorectal motility. Since noradrenergic neurons derived from the pons, especially A5, 6 and 7 regions^5^,^7^,^8^ is one of the major components of the descending inhibitory pathway, the present findings are related to the third hypothesis mentioned above. Considering that pain sensation, which is greater in IBS patients due to visceral hypersensitivity^1^,^2^, activates the descending inhibitory pathway from the brain stem^5^,^6^,^7^, our findings may provide a rational explanation of the concurrent appearance of the abdominal pain and disturbed bowel habits. The notion is supported by the results of a study showing that increases in neural input of a noxious signal into the spinal cord cause a persistent increase in the concentration of spinal NA in rats^9^. Accordingly, it is proposed that abdominal pain promotes motility disorder of the colorectum through activation of descending noradrenergic neurons. It should be noted, however, this proposal does not necessarily mean all pain-related motility disorders are attributable to altered descending inhibitory pathways. It is apparent that interaction between sensitized peripheral visceral afferents and enteric neurons at the peripheral level and/or relationship between central sensitization and descending excitatory and inhibitory pathways need to be addressed in further experiments.

In some IBS patients, spinal hypersensitivity (e.g., expansion of the visceral-somatic referral area) is observed. It is thought that this phenomenon is due to interruption of descending inhibitory pathways^2^,^21^. Interruption of descending inhibitory pathways may also be related to colorectal dysmotility because of the loss of stimulatory effects from the spinal cord via extrinsic nerves. The disordered defecation in IBS patients is known to be heterogeneous and is usually subdivided into diarrhea predominant, constipation predominant and mixed types^3^. We speculate that variation in activation/deactivation states of descending inhibitory pathways is associated with the diversity of disturbed bowel habits.

In summary, we have shown that NA acting on neurons at the lumbosacral defecation center causes propulsive motility of the colorectum in rats. Considering that visceral pain activates descending inhibitory pathways including noradrenergic neurons^5^,^6^, our results provide a rationale for the concurrent appearance of chronic abdominal pain and colonic motility disorder in IBS patients. To our knowledge, this is the first study to reveal underlying mechanisms of the etiological relationship between these symptoms. Our results provide a new insight into the etiology of IBS and suggest a novel target for treatment of disturbed bowel habits.

## Methods

### Animals

Male Sprague-Dawley rats (Japan SLC, Inc., Shizuoka, Japan) weighing 300–450 g were used. The rats were maintained in plastic cages at 22 °C with a 12 : 12 h light : dark cycle (light on 06:00–18:00 h) and they were supplied with both laboratory chow (MF, Oriental Yeast Co., Ltd., Tokyo, Japan) and water *ad libitum* prior to experiments. The experimental procedures were performed according to the guidelines for the care and use of laboratory animals approved by the Animal Care and Use Committee of Gifu University (permission numbers: 12121 and 13076).

### Recording of colorectal motility

The procedures for recording colorectal motility were based on those described previously^12^,^22^,^23^. Sedation was achieved with ketamine hydrochloride (50 mg/kg, i.m.), followed by anesthesia with alpha-chloralose (60 mg/kg, into the tail vein). The femoral artery was cannulated and anesthesia was maintained by intra-arterial infusion of alpha-chloralose (10–20 mg/kg/h) combined with ketamine hydrochloride (3–5 mg/kg/h) in 0.9% saline. The arterial cannula was connected to a pressure transducer for monitoring arterial blood pressure. Body temperature was maintained at 36–37 °C by a heating blanket (Homeothermic Blanket System, Harvard Apparatus, Holliston, MA, USA) throughout experiments. At the completion of the experiments, rats were immediately killed by intraperitoneal injection of a lethal dose of sodium pentobarbitone (100 mg/kg) while they were still under anesthesia.

The colorectum of each anesthetized rat was cannulated both in the region of the distal colon behind the bladder and at the anus, and then the body wall was closed around the oral cannula. The oral cannula was connected to a Mariotte bottle filled with warm saline kept at 37 °C and the aboral cannula was connected to a pressure transducer and a fluid outlet through a one-way valve for measurements of intraluminal pressure of the colorectum and expelled fluid volume, respectively. The basal level of intraluminal pressure was maintained at 3–5 mmHg by adjusting the heights of the Mariotte bottle and outlet tube. Expelled fluid from the aboral cannula was collected in a cylinder positioned beneath the fluid outlet and measured with a force transducer.

For application of drugs to the lumbosacral spinal cord, a 30-gauge needle connected to a polyethylene tube was inserted between L1 and L2 vertebrae from the dorsal surface, until tail flick appeared (L1-L2 corresponding to spinal cord level L6-S1 in the rat). The cannula was secured in place with instant adhesive (Aron Alpha Extra; Toagosei, Co., Ltd., Tokyo, Japan) to create a tight seal at the point of cannulation. There was no cerebrospinal fluid leak. All the drugs used in the present experiments, except for those for anesthesia, were intrathecally applied via the cannula positioned at the L6-S1 spinal cord level. For intravenous injection of atropine, the femoral vein was isolated and cannulated.

When the spinal cord was transected at T8 during the recording session, the region of the T8 vertebra was exposed by laminectomy and the spinal cord was transected with micro scissors^24^,^25^. In some series of experiments, the sympathetic colonic nerves or parasympathetic pelvic nerves were cut before the colorectal cannulation^26^,^27^.

After the surgical operation for recording of colorectal motility, rats were kept for about 1 hour to allow basal colorectal motility and blood pressure to stabilize.

### Reagents

The following compounds were used: alpha-chloralose (Nacalai Tesque, Inc., Kyoto, Japan), ketamine hydrochloride (Daiichi Sankyo Co., Ltd., Tokyo, Japan), NA, phenylephrine hydrochloride, xylazine hydrochloride, isoproterenol hydrochloride, prazosin hydrochloride, atropine sulfate salt monohydrate and tetrodotoxin (Sigma, St Louis, MO, USA). All drugs except prazosin and alpha-chloralose were dissolved in saline. Prazosin was dissolved in 20% dimethyl sulfoxide (Nacalai Tesque), and alpha-chloralose was solubilized with 10% 2-hydroxypropyl-beta-cyclodextrin (Wako Pure Chemical Industries, Ltd., Osaka, Japan) and then made up with 0.9% saline for infusion.

### Statistical analyses

Data are expressed as means ± SD. Statistical analyses were performed using paired, 2-tailed Student’s *t* tests except analysis of dose dependency. One-way ANOVA was used for analysis of dose dependency. *P*-values < 0.05 were considered to be statistically significant. Noradrenaline-induced responses were quantified using data obtained for an initial period of 5 min after the beginning of their appearance. When responses did not appear, data calculation was started 5 min after drug administration. For counting the number of contractions, all pressure increases of more than 2 mmHg above baseline were included.

## Additional Information

**How to cite this article**: Naitou, K. *et al.* Colokinetic effect of noradrenaline in the spinal defecation center: implication for motility disorders. *Sci. Rep.* **5**, 12623; doi: 10.1038/srep12623 (2015).
